# Alcohol consumption and cigarette smoking: a health-risk behaviour among secondary school learners in South Africa

**DOI:** 10.4314/ahs.v22i1.30

**Published:** 2022-03

**Authors:** Nonhlanhla P L Zwane, Ntsieni S Mashau, Violet K Moselakgomo

**Affiliations:** 1 Department of Public Health, School of Health Sciences, University of Venda, Thohoyandou, South Africa; 2 Centre for Biokinetics, Recreation and sports Science, School of Health Sciences, University of Venda, Thohoyandou, South Africa

**Keywords:** Alcohol consumption, Health-risks, Smoking, Secondary School Learners, Tobacco

## Abstract

**Background:**

Health-risk behaviours such as tobacco smoking and alcohol are now identified among adolescents in most of the secondary schools of South Africa.

**Objective:**

The study investigated the prevalence of smoking and alcohol use as health risk behaviours among secondary school learners in Thembisile Hani municipality of Mpumalanga province in South Africa.

**Methods:**

A quantitative descriptive research design was used for the study. A simple random sampling was employed in the selection of schools and proportional stratified sampling was used to select learners from each school according to grades. Closed-ended questionnaires were used to collect data from learners in their schools. Data were analysed using SPSS version 26.0.

**Results:**

Out of 385 learners, 64.4% have drank alcohol whilst 64.7% have smoked cigarette in the school premises. The socio-economic status of many learners such as the employment and income of the family was found to be one of the factors exposing them to alcohol and tobacco use.

**Conclusions:**

There was high prevalence of alcohol use and smoking among learners in secondary schools. Excessive use of alcohol and smoking could affect the health of learners in their late life and therefore community collaboration to curb the problem is crucial.

## Introduction

The global prevalence of chronic diseases due to these health risk factors has increased dramatically in recentyears causing 60% of the 56.5 million reported deaths globally each year^1^. It estimated that 80% of all chronic disease-related deaths such as unhealthy diets, caloric excess, inactivity, andobesity occur in low to middle income countries and are associated with unhealthy behaviours^2^. An international survey by WHO1 on health behaviours among school-aged children showed that overweight or obesity was associated with substance use such as frequent smoking, cannabis use, and drinking, primarily amongst youths. In youths, and particularly amongst boys aged fifteen years or older, obesity was associated with the chances of carrying a weapon, smoking, drinking, marijuana use, unsafe sex risky behaviours such as use of hard drugs, which include cocaine and injection drugs, as represented in a different cluster that included fighting and carrying a weapon amongst white schools in America^3^.

The association between cigarette smoking and health-related behaviours among Chinese School-aged adolescents; smoking is not only a modifiable health-related behavior that can have negative impacts on the physical development of adolescents^4^.

Adolescent smokers are at increased risk for atherosclerosis, asthma and have worse bone mineral density. Smoking is also reported to be linked to lifestyle behaviours, involving diet, as well as alcohol consumption, physical activity, and sleep^4^.

Nicotine is highly addictive, but surveys indicate that almost 70% of US and UK smokers would like to stop smoking^1^. Although many smokers attempt to give up on their own, advice from a health professional increases the chances of quitting. Since 2016 there were 3.5 billion Internet users worldwide, making the Internet a potential platform to help people quit smoking. Moreover, the Palestinian Youth Health Risk Study shows that generally there was a low but not insignificant prevalence of most health-risk behaviours among young people, including alcohol use, drug use, and sexual activity before marriage^5^. The Department of Social Development South Africa has intensified its efforts and implanted a national Anti-drugs Programme which aims to prevent and treat substance abuse by schools^6^. This Anti-drugs Programme was intended to declare all schools drug free zones. In other words, it means that no substance abuse, possession of illegal drugs or alcohol consumption is allowed on school premises.

From a South African perspective, alcohol is particularly attractive to the youth as it is considered a sign of maturity or adulthood. Thus, inappropriate alcohol use among adolescents and youths has been associated with significant behavioural problems, such as aggressiveness or violent behavior, and lethal events, self-injuries, and suicide8. However, not only do Non Communicable Diseases (NCDs) constitute a public health concern but they are also an economic challenge in South Africa1. In South Africa, NCDs are estimated to account for 51% of all deaths, of which 19% are from cardiovascular diseases, 10% from cancers, 4% from chronic respiratory diseases^1^. In Gauteng province of South Africa, alcohol consumption was found to be more common among men and it was estimated that six in 10 men aged 15 and older have consumed alcohol. Five percent of women were reported to be at risk of alcohol consumption and 3% were at the highest risk of developing signs and symptoms of drinking problems^9^.

Several studies revealed that the reason behind the risk behaviours among youths in most countries is that they are undergoing a developmental transition in a rapidly changing social, economic, political and emotional climate^9^,^10^,^12^. Therefore, this volatile environment in which the society is to survive can be a breeding ground for the development of a very complex set of health-risk behaviours that may be intertwined with one another. Furthermore, Al Mojamad^11^ reiterates that adolescence is a transitional stage of physical and psychological human development occurring during the period from puberty to legal adulthood and while significant development occurs during the teen years, full maturity is by no means complete. Decision-making and future-oriented thinking are not fully developed and for these various reasons, the teen years can be a stressful and fragile time, making adolescents more susceptible to engaging in risky behaviours and be unable to weigh their risks and benefits^12^.

Therefore, this paper provide insight to help and inform policymakers to reform or review and implement better the current policy of Integrated School Health Policy on health promotion. This study may also assist the Department of Education and Department of Health to strengthen and promote health education strategies on healthy lifestyle modification. Insight from this may also benefit the learners through encouraging and promoting healthy lifestyle behaviours and decrease risks of getting illnesses and diseases associated with tobacco smoking and alcohol abuse throughout their lives.

## Methods

### Study design and population

A quantitative research approach was used to investigate the health risk behaviours of learners attending secondary schools in Thembisile Hani Municipality in Mpumalanga Province with specificity to tobacco use and alcohol use. We have used the cross-sectional survey design to describe the prevalence of alcohol and smoking as health risk behaviour among secondary school learners. The study was conducted in selected secondary schools of Kwaggafontein East Circuit in Thembisile Hani Municipality. The study was conducted in selected secondary schools of Kwaggafontein East Circuit in Thembisile Hani Municipality. Thembisile Hani Municipality is situated within the Nkangala district in the Mpumalanga Province of South Africa. The area has plenty of informal settlements, with many illegal taverns or beerhalls located near schools. As a result of limited or no recreational facilities around the informal settlements, teenagers are always seen roaming in groups along streets which is a risk factor for boredom and therefore they end up engaging in health risk behaviours such as alcohol use and smoking.

The population was comprised of all learners attending secondary schools in the Nkangala District of Mpumalanga Province and the accessible population was all learners of Kwaggafontein East circuit secondary schools. A purposive sampling technique was used to select one circuit, which was comprised of eight schools with an estimation of 3664 pupils. A sample size of 361 was calculated using the Slovin`s formula. The calculated sample size was increased by 36 to 397 to accommodate non-responses. A stratified sampling was used according to grades and gender of participants; thereafter random sampling was used to select the proportional sample in each stratum. Inclusion criteria for this study were all learners aged 15 to 20 years old. These learners should have been in grades 10–12 and registered in all the secondary schools under the Kwaggafontein East circuit of Mpumalanga province in South Africa.

### Data collection and analysis

Data were collected using a self-administered questionnaire. The questionnaire consisted of close-ended questions with some phrased in a Likert scale format. A reliability test was done using Cronbach`s Alpha (α) to test the internal consistency of the data collection tool. The Cronbach's alpha coefficient result of 0.8 was achieved. Both content and face validation were employed to ensure the validity of the instrument. Questionnaires were distributed to the sampled participants of each grade and it took 25 to 30 minutes to complete and questionnaires completed questionnaires were collected on the same day. Out of 397 questionnaires, only 12 were spoiled and 385 were fully completed, which is 97% response rate. The data were coded and captured using Microsoft Excel (2016) and exported to Statistical Package for Social Science Software (SPSS) version 26.0 for analysis. The Chi-square test were used to determine the relationship between variables.

### Ethical considerations

#### Ethical approval was obtained from the Research

Ethics Committee of the University of Venda (SHS/19/PH/18/2708) in 2019. A written permission to collect data from the secondary schools of Kwaggafontein circuit was obtained. A detailed written information about the study was provided to participants to read and understand their rights to participate in the study. Informed written consent forms for participants who were 18 years and above were obtained. However, learners who were younger than 18 years were given assent forms to sign and consent was obtained from their parents after they had been fully informed about what was expected of the participants and the nature of the study. Anonymity and confidentiality were ensured and the names of participants were not written on the questionnaire, instead codes were used.

## Results

Table 1 shows the demographic information of participants. Out of the 385 learners, females were in majority because they constituted 55.3% with a minority 45.7% of males. The study was dominated by learners who were between the ages of 16–18 years. However, learners who were17 years old were in majority (43.9%), whilst those who were 20 years old were the least (6.2%) age group in the study. The study was conducted among grade 10–12 learners, however, the grade 11 (33.8%) and 12 (46.2%) had the highest number respectively, with minority (20%) being grade 10.

**Table 1:** Demographic characteristics of participants N=385

	Variables	Frequency (n	Percentage (%)
**Gender**	Female	213	55.3
	Male	172	44.7
	
	**Total**	**385**	**100**
**Age**	16 Years	82	21.3
	17 Years	169	43.9
	18 Years	68	17.7
	19 Years	42	10.9
	20 Years	24	6.2
	**Total**	**385**	**100**
	
**Grades**	Grade 10	77	20.0
	Grade 11	130	33.8
	Grade 12	178	46.2
	**Total**	**385**	**100**

Source: Current study data

As shown in table 2, although the majority (86.2%) of participants indicated that cigarette smoking is dangerous to health, 64.7% of participants indicated that they have smoked cigarette in the school premises whilst. 17.1% have tried to smoke other substances such as dagga, glue, cocaine. The results further showed that 38.4% indicated that it is easy to access smoking substances in their communities

**Table 2:** Smoking and access to substance Statement

	Yes		No		Don't know	
	n	%	n	%	n	%
Smoking substances are easy to get in your community	148	38.4	147	38.2	90	23.4
Cigarette smoking is dangerous to health	332	86.2	18	4.7	35	9.1
I`ve tried smoking substances e.g. marijuana, dagga, cocaine, glue	66	17.1	193	50.1	126	32.7
I`ve smoked cigarette in the school premises	249	64.7	80	20.8	56	14.5
Smoking can cause lung disease such as Asthma, Cancer	207	53.8	134	34.8	44	11.4

Source: Current study data

The study assessed if learners were taught in class about the dangers of smoking. The results revealed that 42% of learners were taught about the dangers of smoking in class whilst 64% indicated that smoking releases stress. As shown in table 3, the majority (64.4%) of participants indicated that they have tried drinking alcohol in their life whilst 27% indicated that they drank alcohol before the age of 14 years. The results further revealed that 57.1% were in trouble at home or school because of alcohol use whilst 71.9% of learners visited a beer hall or bottle store.

**Table 3:** Alcohol consumption among learners

	Yes		No		Don't Know	
Alcohol consumption	n	%	n	%	n	%
Anybody in your family who consumes alcohol	14	3.6	242	62.9	129	33.5
Ever drank alcohol in your life	248	64.4	59	15.3	78	20.3
Have friends or school mate who drinks alcohol	115	29.9	270	70.1	--	--
Ever drank alcohol before 14 years of age	104	27	75	19.5	206	53.5
At least had one drink of alcohol during the past 30 days	42	10.9	112	29.1	231	60
Know that alcohol abuse can cause sicknesses	148	38.4	195	50.6	42	10.6
Drink on special occasions only	60	15.6	309	80.3	16	4.2
Ever been inside the bottle store or tavern	277	71.9	108	28.1	--	--
Ever had been in trouble at school or home because of alcohol abuse	220	57.1	165	42.9	--	--
Taught in any classes about the dangers of excessive alcohol consumption	72	18.7	195	50.6	118	30.6

Source: Current study data

**Table 4:** illustrates the study findings on the age of participants, smoking and tobacco use as a health risk behaviour among learners. The cross-tabulation and the Chi-square test were statistically significant, as it shows that age influences the decision in engaging in smoking. The study findings show that smoking is most prevalent in younger age were self-control is still low (P-0.000, x2-86.253, df-16).

**Table 4:** Cross-tabulation (age of Participants and tobacco use)

df-=16, p=0.000						

		Tried cigarette smoking one or more puffs				
		Strongly Agree	Agree	Unsure	Strongly Disagree	Disagree
**Age of** **Participants**	16 Years	61	0	0	0	21
	17 Years	118	16	17	1	17
	18 Years	68	0	0	0	0
	19 Years	42	0	0	0	0
	20 Years	24	0	0	0	0
	Total	313	16	17	1	38

Source: Current study data

**Table 5:** illustrates the study findings on the gender of participants and alcohol consumption as a health risk behaviour among learners. The cross-tabulation and the Chi-square test were statistically significant, as it shows that alcohol consumption increases with the grades of learners, wherein grade 12 were the ones who were the majority who consume alcohol (P-0.000, x2-187.703).

**Table 5:** Grades and alcohol consumption

df-4, p-0.000					

		Ever drank alcohol in your life?			Total
		Yes	No	Don't know	
**Grades**	Grade 10	62	0	15	77
	Grade 11	27	57	46	130
	Grade 12	159	2	17	178
Total		248	59	78	385

Source: Current study data

## Discussion

About 53.5% of the learners in this study started drinking on and before the age of 14 years of age and most of them had a high frequency of drinks just before the study was conducted. This might be due to the fact that at 14 years, learners are still young and immature to make responsible decisions. A slightly different longitudinal study in East Asia13 indicated gender differences that highly active teenage girls were less likely to start smoking during the study, than the low active ones. In boys, however, no difference occurred between the high and low active groups, while the highly active boys were twice as likely to start drinking. In terms of location which was not covered by this study, the highest level of alcohol consumption occurs in the developed world, this fact is not surprising since the history of alcoholic beverages is linked to the history of mankind in which for centuries, and alcohol consumption has been part of the African culture and society.

Given that drinking alcohol is a social activity, embedded today in traditional and sociocultural contexts there is a social mindset that the main reason for alcohol consumption is its ability to produce positive moods and stress-relieving effects. The present study therefore established that this societal perception might be a contributory factor to high alcohol consumption among learners and some studies has attested that alcohol consumption among learners contributed to high prevalence of suicidal ideation which occurred in the highly active boys and girls^13^,^15^. In the USA it was found that the majority of adolescents under the age of 18 have consumed alcohol, although the minimum legal drinking age is 21 years old.

The present study has indicated that alcohol consumption increases with grades, as grade 12 were seen to be drinking a lot than grade 10 however, in contrary15 no substantial differences existed among various sociodemographic subgroups concerning drinking rates. However, alcohol consumption generally is lowest among Africans and highest among Whites. Moreover, alcohol consumption increases sharply throughout adolescence due to various attitudinal and behavioural factors, such as religious involvement, truancy, and average grade level, which also influence adolescents' drinking behaviours.

The present study did not document and present a detailed account of peer influence as a central cause of alcohol consumption as^14^ who consistently find that adolescents who associate with drinking peers have less success with quitting. The high alcohol drinking rates among youth indicate that addressing peer influences may be particularly important with this population. It is crucial because adolescents strongly identify with their friends and peers, a phenomenon which is central to an adolescent's development of a self-image distinct from one's family^13^. Therefore, the role of peers in adolescent smoking can be understood as part of an adolescent's social identity and peer selection rather than solely as peer pressure.

A similar study to the above assertion shows that during adolescents' neurological development is not complete until the early twenties, wherein decision-making and future-oriented thinking are not fully developed^16^. Thus, while teens are entering into adult roles and while they may physically appear to be mature, teens might not be fully equipped to deal with these new tasks and challenges and for these various reasons. Therefore, the teen years can be an especially stressful and fragile time, making adolescents more susceptible to engaging in risky behaviours and be unable to weigh their risks and benefits.

The results of the present study established that most learners came from drinking families and most of these learners have been visiting the taverns around the community. Therefore, a family history of alcoholism is an important risk factor associated with using alcohol for the first time at an earlier age. Consistently, Maslowsky, Schulenberg, Chiodo, et al.^15^ attested that having a family history of alcohol drinking problems is associated with greater underage drinking and greater frequency of alcohol-use problems. Several studies have reported that only the father's drinking habit has a direct effect on adolescent drinking, however, further research is needed to clarify the real impact of parental drinking on adolescent drinking as it is known that alcohol abuse tends to be repeated within families^11^,^13^,^17^. Family and environment are positively related to drug use among youth. It is believed that the parenting style significantly protects against adolescent drinking behaviour with both indulgent and authoritative parenting styles have been suspected to be a major source of influence on protection against adolescent substance use.

The results of the present study indicate that most pupils at a tender age are engaging in smoking or tobacco use and alcohol consumption, which puts them at risk of getting life-threatening diseases. Other related studies by Kerr, Lui and Williams et al.^13^ and Brown^18^, detailed that an increased psycho-social distress results in smoking or tobacco abuse in sub-Saharan youth, may also lead to suicidal ideation and other destructive emotional experiences such as sadness and hopelessness. A slightly different longitudinal study in East Asia^13^ indicated gender differences in that highly active teenage girls were less likely to start smoking during the study, than less active ones. In boys, however, no difference occurred between the levels of activeness, while the highly active boys were twice as likely to start drinking.

## Limitations of the study

This study posed some limitations, which should be borne in mind when interpreting the results. The sample in this study was relatively small, and although randomly selected, it only represents one Municipality in Mpumalanga. The smaller urbanized centres may reflect different profiles. The population involved were those attending secondary schools. As the questionnaires were based on self-assessment, it was possible that participants either over- or underscored themselves. Despite these limitations, the current study is probably the one of the researches projects that assessed toffers some insights into the relationship existing between different health risk behaviours among Mpumalanga youth. It confirms the complexity of Mpumalanga adolescents' health risk behaviours and shows the necessity of future research in this area in order to develop appropriate health promotion interventions and it can be used for comparison to other places.

## Conclusions

This paper highlights that a large number of young learners at the Thembisile Hani Municipality are drinking alcohol and smoking tobacco, and this may contribute to adverse health outcomes and disruptions amongst others or the community. The socioeconomic status, which entailed inadequate or no income and unemployment of parents, was a pre-disposing factor to health-risk behaviors such as alcohol use and tobacco smoking among learners.

## Recommendations

A collaborative effort between community stakeholders, civil structures and the government departments should engage in addressing the problem of health-risk behaviours among teenagers and youth. Recreational facilities in schools should be established and learners should be actively involved in sports activities. Learners should be encouraged to participate in the different sports codes in order to keep them busy and therefore discourage boredom. The minimum legal drinking age should be increased to 21 years.
